# Vehicular Traffic Flow Analysis and Minimize the Vehicle Queue Waiting Time Using Signal Distribution Control Algorithm

**DOI:** 10.3390/s23156819

**Published:** 2023-07-31

**Authors:** Srinivasagam Solaiappan, Bharathi Ramesh Kumar, N. Anbazhagan, Yooseung Song, Gyanendra Prasad Joshi, Woong Cho

**Affiliations:** 1 Department of Mathematics, Anna University, University College of Engineering, Ramanathapuram 623513, Tamilnadu, India; solaimagita@gmail.com; 2 Department of Mathematics, Vel Tech Rangarajan Dr. Sagunthala R&D Institute of Science and Technology, Chennai 600062, Tamilnadu, India; brameshkumar@veltech.edu.in; 3 Department of Mathematics, Alagappa University, Karaikudi 630003, Tamilnadu, India; anbazhagann@alagappauniversity.ac.in; 4 Electronics and Telecommunications Research Institute, Daejeon 34129, Republic of Korea; yssong00@etri.re.kr; 5 Department of Computer Science and Engineering, Sejong University, Seoul 05006, Republic of Korea; 6 Department of Electronics, Information and Communication Engineering, Kangwon National University, Samcheok-si, Gangwon State 25913, Republic of Korea

**Keywords:** vehicular traffic congestion, vehicular traffic classification, vehicular traffic detection, signal parameters, TSM mode, MATLAB coding, data collection interruption Junction

## Abstract

The real-time vehicular traffic system is an integral part of the urban vehicular traffic system, which provides effective traffic signal control for a large multifaceted traffic network and is a highly challenging distributed control problem. Coordinating vehicular traffic enables the network model to deliver an efficient service flow. Consider that there are four lanes of vehicular traffic in this situation, allowing parallel vehicle movements to occur without causing an accident. In this instance, the vehicular system’s control parameters are time and vehicle volume. In this work, vehicular traffic flow is examined, and an algorithm to estimate vehicle waiting time in each direction is estimated. The effectiveness of the proposed vehicle traffic signal distribution control system by comparing the experimental results with a real-time vehicular traffic system is verified. This is also illustrated numerically.

## 1. Introduction

In day to day life, everyone would have encountered the problems of vehicular traffic overcrowding and anticipate prompt assistance. Nowadays, growing urbanization causes traffic jams, which necessitates a solution to operate our traffic vehicle flow system with the greatest flow efficiency. The system’s effectiveness depends on how easily it can be used and accurately forecast vehicular traffic. The minimal waiting time and cost were used as key parameters in the literature for all the vehicular traffic signal flow models. The vehicular signal control system synchronizes the time in all signal directions to reduce vehicle waiting time and overall delays. The major issues for the traffic queue in the current traffic model may be the length of the road junction, the volume of vehicle traffic, turn %, and time calculation. The traffic congestion will not be a constant issue during peak hours and other times. The placement of traffic lights at the crossroad intersection is intended to regularize the traffic flow. This condition must be met in order to decrease the total waiting time in all directions will be the first step in all suggested algorithms based on the total vehicle flow time in each direction. Several researchers have come to various conclusions regarding the above-mentioned problems. The dynamic vehicle traffic signal plan management system using the reflection principle [1] offered a consistent and unique solution. This model illustrates the vehicle traffic flow stability using extensively utilized proportional distribution controllers. The ideal vehicle control structure for the multi-agent model of a vehicle single-panel system was created by [2]. The control system function, augmented by the (GPVE), simulates the traffic parameters in this model, which are optimized using a genetic algorithm. In order to enhance the performance and examine the diversity quality, by using the memtic method, an urban vehicle traffic management system was suggested [3,4]. A Monte Carlo-based approach to signal timing scheme and the elimination of the connection system was designed [5]. In this approach, the cost value was used for the objective function to produce the number of vehicles serviced per hour. In [6], the infrastructure employing a support vehicle network has been designed, to reduce traffic congestion and create a smart vehicular traffic system. In this system, the priority of the traffic signal characteristics is calculated, along with the environmental driving conditions. A self-adaptive signal control algorithm for the adjustment of signal parameters of the transport requirements with seasonal changes has been designed [7], to provide a radical solution for eliminating traffic congestion. An evolutionary algorithm-based system for real-time vehicle traffic improvement was discussed by considering traffic signal control and fixed time [8]. The vehicle line lengths discovered was lower than the predetermined signal-rated time, from which the interruption has been greatly decreased. Using swarm optimization strategies [9], enhanced the traffic signal control issue and examined traffic model performance mathematically and statistically relating to the application with reliable traffic data. A novel lane bypass algorithm for route diversion using a heuristic search for the efficient flow of traffic on the metropolitan road network has been presented [10]. The optimization of time parameters was done using genetic algorithms for traffic control signals was discussed [11], and examined the effectiveness of the system at cross road intersections. An open Jackson queuing model using an area of traffic congestion was designed [12], to determine the vehicle waiting time in the traffic network flow model. In all traffic flow directions, this model was implemented to analyze the waiting time from the range of potential service rates. The service rate combination was selected with the minimum waiting time to measure the accuracy of the model. A framework for effective traffic prediction and the best alternative route selection was developed [13] using the (HBI-LSTM) and (HV-ABC) approaches to create an effective road traffic control forecast framework. The YOLOV4 algorithm was used [14] for the detection and recognition of traffic lights. A shallow feature augmentation method and a bounding box uncertainty prediction mechanism were used in this model. By using the improved YOLOV4 algorithm, the recognition of small targets and traffic light detection was significantly increased. The reliability of YOLOV4 is further increased by identifying traffic lights, which forecast the movement trajectory and status of the traffic light in relation to the vehicles.

The adjusted Controlled Pass-By(CPB) technique is used for assessing the noise and determining the overall acoustic performance produced by road infrastructure [15]. A number of technical changes are outlined, including the delineation of markings directly on the road surface to serve as a driver’s guide, support the repeatability of the trajectory during each passage, and help drivers avoid unwelcome road features like speed bumps and manhole covers. By examining the timing of the peak levels in the octave frequency band relative to the position of the passage’s metric, the proposed model verified a method to identify outliers in the log-linear fit. Using the captured images and convolutional neural network, ref. [16] has developed a quick and inexpensive system that generates the height maps of the road surface. The sample data were gathered using the closed, four-light, photometric theory as a foundation, followed by a transformation matrix. The measured laser profiles served as the ground truth; the image intensities and light directions per pixel were provided as input. With two key contributions, the suggested model using CNN offers an alternate method of evaluating road surfaces. The novelty of this method is that the surface reconstruction does not require knowledge in the areas of photometry, computer vision, or numerical optimization. Also, it needs a consumer camera rather than expert equipment to achieve the associated economic decrease. The Unattended Statistical Pass-By (U-SPB) model [17], which has been adapted to the SPB-ISO in an urban context, was proposed to measure urban road traffic noise. This model measures the noise performance of passing vehicles without the operators. The testing, validation and application of U-SPB was carried out at different locations. The adaptive traffic flow and collision avoidance technique for vehicle platoons based on CACC has been proposed [18], for the evolution of platoons in order to prevent unclear circumstances. By joining two small platoons together, the merge and the join maneuvers are used to cut down on overall travel time and road overhead. According to the comparison study, combining small platoons can reduce the amount of time a platoon must travel. A dynamic traffic interval methodology that divides traffic flow into low, medium, high, and extremely high volumes has been proposed [19]. Based on real-time traffic data, including information about pedestrians and vehicles, the model modifies traffic light intervals. When compared to the fixed time and semi-dynamic traffic light control methods, this dynamic method reduces the vehicle waiting time from 12% to 27% and pedestrians’ waiting time from 9% to 23% at an intersection. This method is not applicable at every intersection because it depends on the service that consumers desire. The minimum vehicle delay problem, referred to as the vehicle signal control performance of the vehicle traffic management system at cross-sectional area structures, is critical for the urban road transport network system. The specific considerations discussed by the researchers in the above literature are mainly about fixed signal timing and traffic parameter allocation. The present fixed signal timing flow model will not efficiently manage the traffic flow during peak hours and other timings. As a result, the vehicle queue length increases during peak hours, which leads to issues like congested intersections, lost time, environmental pollution, etc. The queue length can be reduced by using a dynamic signaling system instead of a conventional fixed signaling model. The traffic signal waiting time can be effectively managed by calculating the number of vehicles arriving towards the cross junction. In this article, the vehicular traffic flow analysis has been performed and a signal distribution control algorithm is proposed to reduce the vehicle waiting time at the cross junctions. The aim of the proposed model is to generate dynamic signal time to suggest the equal management of the vehicles with minimum waiting time at all phases of the intersection. The novelty of the proposed method is that this traffic signal model calculates the pass and stop signaling time duration based on the number of vehicles arriving from all directions towards the junction. If the time flow of vehicle arrival in a particular direction is less than the assigned signal time duration, then the leftover time is adjusted to the next consecutive directions.

The performance analysis of the proposed dynamic signal timing system shows a better performance when compared with conventional signaling system using the following parameters.

- Service provided for a number of vehicles per cycle length
- Surplus rate of vehicle overflow per cycle length
- Amount of vehicle waiting time for each cycle length
- Overflow vehicle count
- Waiting time overflow
- Reduction in total waiting time overflow

The remaining sections of the article are organized as follows. Section 2 discusses the traffic signal control parameters. Section 3 provides the overview of traffic vehicle flow direction with the mathematical model and algorithm of the proposed method. In Section 4, the proposed method is proved with the numerical example. In Section 5, the simulation results were discussed. Section 6 concludes the article.

## 2. Traffic Signal Control Parameters

The general characteristics of the traffic vehicle signal control system are covered in this section. It is used to rate the effectiveness of signal timing in terms of operations and safety. A general review of the system and user characteristics affecting signal timing is presented. Capacity is the maximum rate at which vehicles can pass through the intersection under prevailing conditions. Clearance lost time means the time, in seconds, between signal phases during which an intersection is not used by any critical movements. Control Delay is the amount of additional travel time experienced by a user attributable to a control device. Critical movement analysis is a simplified technique for estimating phasing needs and signal timing parameters. The delay time is the additional travel time experienced by a driver, passenger, or pedestrian. Effective green time is the time during which a given traffic movement or set of movements may proceed; it is equal to the cycle length minus the effective red time. Flow rate is considered as the equivalent hourly rate at which vehicles, bicycles, or persons pass a point on a lane, roadway, or other traffic way, computed as the number of vehicles, bicycles, or persons passing the point, divided by the time interval (usually less than 1 h) in which they pass; expressed as vehicles, bicycles, or persons per hour. Level of service is a qualitative measure describing operational conditions within a traffic stream based on service measures such as speed and travel time, freedom to maneuver, traffic interruptions, comfort, and convenience. Lost Time is the portion of time at the beginning of each green period and a portion of each yellow change plus red clearance period that is not usable by vehicles. The saturation Flow Rate is the equivalent hourly rate at which vehicles can traverse an intersection approach under prevailing conditions, assuming a constant green indication at all times and no loss time, in vehicles per hour or vehicles per hour per lane. Start-up lost time is the additional time, in seconds, consumed by the first few vehicles in a queue at a signalized intersection above and beyond the saturation headway due to the need to react to the initiation of the green phase and to accelerate to a steady flow condition. Stopped Delay is the measurement of the aggregate sum of stopped vehicles for a particular time interval divided by the total entering volume for that movement. Total delay is the sum of all components of delay for any lane group, including control delay, geometric delay, and incident delay. The total elapsed time spent traversing a specified distance. The average travel time represents the average of the runs for a particular link or corridor. Refs. [20,21] discussed that the discrete traffic signal controllers operate independently of the rest of the signal controllers. An integrated signal controller means that most signal controllers are synchronized with each other. The isolated junction is too far from adjacent connections; the signal direction is not amplified in the functions. Arterial Junction provides vehicle traffic signal coordination in the first lane. A network assembly is supplied to each junction, resulting in small splits and stable signal junctions typically produced in densely populated urban regions. The link is what joins the two junctions together. Two consecutive periods have regarded the green signal phase. A red signal typically indicates a wait and line at a crossroads junction. The time needed for a full-phase revolution is called the rotational length. It is intended for dynamic situations where the volume of traffic changes from cycle to cycle. Based on changing traffic conditions, the method provides a unique control for each cycle. The management plans to employ a dynamic process that explicitly takes demand and present and projected queue lengths into account when designing signal control settings. The proposed method is significant to the traffic light control management techniques. It’s very helpful to the future traffic management plans are generated and evaluated simultaneously over both space and time by the approach.

## 3. Traffic Vehicle Flow Direction

**Figure 1:**
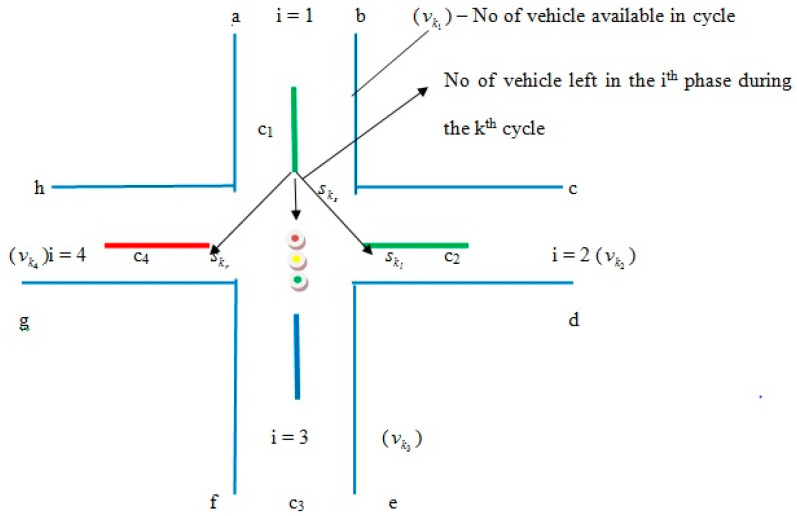
Vehicle Path Flow.

The suggested method of optimization for the best traffic vehicle light rotation schemes is described in this section. The objective function, solution code, and optimization procedure are all covered in this section. The self-propelled design and planning algorithm, which provides centralized comfort to the approaching vehicle at each stage, is taken into account in the proposed traffic signal processing service rate. This process’s two crucial aspects are shortening the cyclic queue length and monitoring system performance. The proposed method achieved the correct signal path. Therefore, effort has been made to increase the system performance and traffic flow rate. See Figure 1.

The TSC system measures the average vehicle flow rate and service rate per unit. However, the length of the preceding cycle’s backlog was not taken into account. This program analyses signal device solutions for various transportation needs and is easily adjusted for the TMS. The algorithm’s approach increases device control parameters compared to those used before the overflow service period and decreases the vehicle line-up. The overall attendance and service ratio has been developed to guarantee a consistent measurement of the distribution of green signals for the traffic management system. The traffic signal lights for the traffic signal are in control of the labeled points here (a, b, c, d, e, f, g and h). The traffic flow path, expressed by the node, is called edges because two edges connect the path flow and do not crash the flows together. Initially identify the possible direction allowed to enter the intersection area such as (i) current index of the left-turned flow it should follow the intersects with the current flow, (g) (ii) flow index (h & b) it does not intersect the flow of (c & e). Following are the different signal flow directions: a & g may not run parallel, although the flow h & C with d & e it may be run in parallel) (a) Marking various initial flows (b) Making the availability of prior assumptions, such as the flow of left turn signal (c) Whether the current left-turned signal flow has the same end vertices. (iv) Express each flow with its corresponding network node (v) when two nodes are connected by an edge on a signal network, the flow is considered sequential if the two connected nodes are well-matched. Nodes at intersections serve as connecting points for two additional intersecting roads in traffic management networks. They are an integral aspect of the system. The elimination of intersection junctions will reduce driver conflict and offer unrestricted security for traffic vehicle flow. Numerous metrics are included in the traffic management system in order to analyze the traffic signal system. It is referred to as flooded flow traffic when the performance of an intersection junction between (i) vehicles is optimized. Although the flow is not the same for intersection roads, the vehicle capacity can be based on the hourly limits of the intersection roads because the following flow rate was slightly inflated: (a) Traffic control measures. (b) Increase the likelihood at each intersection junction (c) radius (d) ran into enter from the opposite direction and turns to the right. Phase: It can be given to the ideal assignment of traffic flow movements and is a component of the traffic signal light cycle length. It will be split up into a number of sub-flows. Traffic flow direction must be followed by the labeled points as follows: (1) Flow a = (b, h, e, f), (2) Flow b = (a, h, d), (3) Flow h = (a, b, g, d, e, f) (4) Flow g = (h, e, f), (5) Flow c = (h, d, e), (6) e = (a, h, g, e). The character’s premise for the crossroads junction is the following: (1) does not transition smoothly from a right turn to a left turn. Since the flow moving time has zero waiting time, (2) the last two left flow nodes (a and e) are travelling in accordance with the traffic signal. Here, the straight flow nodes are (b, g, d, and g), whereas (a, h, & e) are three-turn left flows, and (c) is a one-turn right flow. If flows connect the following sites in a parallel fashion, the node joins two pathways. In order to prevent accidents and reduce overall waiting times, traffic signal intersection road traffic management systems should be organized for optimal control.

### 3.1. Formulation of the Traffic Management Procedure

In this section presented the proposed algorithm, system resource constraints, Pseudo Algorithm and optimization procedure are all covered. Generally, a traffic light signal system considers the arrival and service rates. The overflow rate and queue length of the previous cycle are not considered. The proposed algorithm has the capability to modify to design and analyze control strategies with different traffic light conditions. Green signal time is calculated based on the arrival and service rates to ensure continuous movement and effective utilization of the green time.

K→Phase index
$C_{i}$→Indicator for the cycle (*i* = 1, 2, 3, 4)
$Dv_{kc}$→Reaching from vehicle *k* during the cycle
$q_{kc}$→The length of the queue or equation at the beginning of the cycle
$Nv_{kc}$→Vehicle number/came in the *k*th phase of the cycle
u→Average vehicle/service provided during the cycle
$Tv_{kc}$→Total number of vehicles in *k*th-indicator of the cycle length
$T_{i}$→Indicator total service time (or) service provided in the *k*th phase for the cycle length
$S_{kc}$→The number of vehicles left (or) provided the service in the *k*th indicator cycle length
P→Provided by the service time/s
$o_{kc}$→The total number of vehicle overflows rates during the *k*th indicator cycle length
$W_{kc}$→The total number of vehicles waiting time during the *k*th indicator during the cycle length
W→Total system waiting time

The entire traffic management system can be considered a “*k*” phase modeling system and provides service to all vehicles arriving during consecutive breaks.

Total signal time per cycle length is equal to the sum of the total signal time in each phase. The service provided in the *k*th phase for the cycle length:

(1) $$T_{c}=\sum_{i=1}^{n}T_{k_{i}}\;where\;T_{k_{i}}=V_{k_{i}}/V_{k}$$

Availability of the number of vehicles per cycle length:

(2) $$V_{k}=\sum_{i=1}^{n}V_{k_{i}}$$

Service provided for a number of vehicles per cycle length is directly proportional to the total service time and total number of vehicles in each direction.

(3) $$S_{k}=\sum_{i=1}^{n}s_{k_{i}}\;where\;s_{k_{i}}=p\times T_{i}\;\&\;p=\mu /T_{c}$$

Surplus rate of vehicle overflow per cycle length is the difference between the total number of vehicle overflow rate and total numbers of vehicles leave the system in each direction.

(4) $$O_{k}=\sum_{i=1}^{n}o_{k_{i}}\;where\;o_{k_{i}}=v_{k_{i}}-s_{k_{i}}$$

The amount of vehicle waiting time for each cycle length is related to the total signal time per cycle length and total vehicle over flow rate in each direction.

(5) $$W=\sum_{k=1}^{m}W_{k\;}\;where\;w_{k_{i}}=o_{k_{i}}\times T_{c}$$

So,

(6) $$\%R=\frac{EW_{kc}-PW_{kc}}{EW_{kc}}\times 100$$

where PW—The total number of vehicles waiting in the proposed manner, EW—Total vehicle waiting time in the existing manner.

### 3.2. Generalized Algorithm of Traffic Signal Control System


(7) $$\mathrm{Step}\;1:\;d\;v_{kc}\leq q_{kc(i,i+1)}+D\;v_{kc(i,i+1)}$$

(8) $$\mathrm{Step}\;2:\;Nv_{kc}\geq D\;v_{kc}\;\;i=1,2...n$$

(9) $$\mathrm{Step}\;3:\;Tv_{kc}=\;sum\;of\;\;step(2)$$

(10) $$\mathrm{Step}\;4:\;T_{i}=N\;v_{kc}/T\;v_{kc}$$

(11) $$\mathrm{Step}\;5:\;s_{kc}=P_{x}T_{c}$$

(12) $$\mathrm{Step}\;6:\;S_{kc}=sum\;\;of\;\;s_{kc_{i}}\;\;i=1,2...$$

(13) $$\mathrm{Step}\;7:\;O_{kc(1,i+1)}=\;\;N\;v_{kc(i,i+1)}-S_{kc(i,i+1)}$$

(14) $$\mathrm{Step}\;8:\;O_{kc}=\sum_{i=1}^{n}O_{kc(i,i+1)}$$

(15) $$\mathrm{Step}\;9:\;W_{kc_{i}}=\left[Nv_{kc(i,i+1)}-S_{kc(i,i+1)}\right]\times \sum_{i=1}^{n}T_{i}$$

(16) $$\mathrm{Step}10:\;W=\sum_{k=1}^{n}W_{kc_{1}}$$

(17) $$\mathrm{Step}\;11:\;\%R=\frac{EN_{kc}-PW_{kc}}{EW_{kc}}\times 100$$

### 3.3. Constraints Expression

- If the *k*th phase system’s successive arrivals for *i* and *i* + 1. The displacement from the (*k* − 1)th phase to the *k*th phase will be made by the vehicle in the (*i* + 1)th stage. In this scenario, the crossroad junction areas’ cycle time and total vehicle interruption of the *k*th phase queue length will be delayed.
- The availability of the *k*-phase index arriving vehicle is greater than the delayed queue length.
- The total number of *k*-phase left over vehicles replacement is multiplied by the average number of arriving vehicles.
- The vehicle waiting time specified in the scheme is equal to the average waiting time of the *k*th indicator phase throughout the cycle.
- The total vehicle surplus ratio equals the number of vehicles that arrive from the *k*th indicator phase over the cyclic period.

### 3.4. PSEUDO-Algorithm

This is Algorithm 1.
**Algorithm 1**Function Traffic_Signal_Control Function Vehicle_to_RSU(Vehicle count, Time) Set Vehicle count to 0 While (Time interval)   FOR each Vehicle and RSU      Do vehicle count = vehicle count + 1   End FOR End While End Function Function RSU_to_Data_Manager(Vehicle count, Time) For 0 to Total_record   Store data   Clean data   Pre_process data End For End Function   Function Signal_Decision(direction, vehicle_count, time)      For 0 to direction + 1         For 0 to time + 1            Signal_time(direction, vehicle_count)            Status = On            control_unit(direction, time, status)      End For   End Function   Function control_unit(direction, time)      For 0 to direction + 1         Signal_status = status      End For   End Function End Function

## 4. Numerical Example

Consider the four-phase intersection, where the available vehicle phases are 100, 150, 350, and 200, respectively. For each cycle, the total signal time and service rate are fixed at 300 s and 600 V/s, respectively, offering the following two vehicles limitless service. Calculate the average waiting time and overflow rate using the proposed algorithm. The outcome is then evaluated in comparison to the current methodology. The primary variables used for in-depth collection and analysis are the number of passing cars, time, intersection phase, day order, and rotation time. $T_{c}=300\;s$, μ=600 V/s and ρ=2 V/s.

**Using the Conventional method**, the vehicle waiting time for each cycle length is obtained. The service provided in the *k*th phase for the cycle length is $T_{i}=75\;\;s,i=1,2,3,4$. The availability of the number of vehicles per cycle length is $v_{k}=800$. Service provided for the number of vehicles per cycle length is $S_{k}=550$. The surplus rate of vehicle overflow per cycle length is $O_{k}=200$. The vehicle waiting time for each phase length is:$w_{k1}=w_{k2}=0$, $w_{k3}=60,000$, $w_{k4}=15,000$. Therefore $W_{k}=75,000\;s$.

**Using the proposed method**, the vehicle waiting time for each length is obtained. The service provided for each cycle length is $T_{1}=(100/800)*300=37.5$, $T_{2}=58.25$, $T_{3}=131.25$, $T_{4}=75$. The number of vehicles available per cycle length $S_{k}=601$. The vehicle overflow surplus rate in each phase is $o_{k1}=100-75=25$, $o_{k2}=150-13=137$, $o_{k3}=300-213=87$ and $o_{k4}=200-150=50$. So, $O_{k}=199$. The vehicle waiting time of each phase length is: $w_{k1}=25*300=7500$, $w_{k2}=37*300=11,100$, $w_{k3}=87*300=26,100$, $w_{k4}=50*300=15,000$, $w_{k}=59,700\;s$.

Result comparison for the proposed and existing method:

$$R=\frac{75,000\;-\;59,700}{75,000}\;\times 100=20.49$$

### 4.1. Computational Method

#### Experimental Setup

The simulation of an urban mobility program, also known as SUMO, which is frequently used to generate a simulated traffic environment that closely resembles any real-time condition, was utilized to establish the experimental setting. Three lanes are used for commuter traffic in this model’s four perpendicular roads scenario. Out of these, the leftmost lane is reserved for left turns, the rightmost lane is fixed for right turns, and the center lane is set aside for through-passing vehicles. With the lengths set at 100 m, an intersection area of 200 m × 200 m was taken into consideration. The vehicle has a set length of 5 m, and a 2 m minimum distance is anticipated between them. The grid size is set to 5 m, resulting in a total of 40 × 40 grids. With a set vehicle arrival time of 1/10 of a second, all the cars come in a random sequence. Two through-pass lanes are available, and traffic moving from the west, east, north, and south bounding to the east, west, south, and north will move at a rate of 2/10 of a second. A flow rate of one tenth of a second will be experienced by the vehicle turning from east, west, south, and north and moving towards south, north, west, and east, respectively. The vehicle can travel through the intersection at a maximum speed of 30 km/h, with the accelerating and decelerating accelerations being calculated at 1.0 and 4.5 m/s^2^ respectively. The dataset from the Solinganur signal station in Tamilnadu, India, is used to test the proposed algorithm. The dataset complies with the open data policy’s requirements and is accessible to the general public. It consists of daily traffic volumes taken at Solinganur every ten minutes. Other important details are sensor placement and flow direction information. The main factors that were carefully gathered and studied included the volume of passing cars, time, intersection phase, day order, and cycle time. Table 1 mentions data collection.

Here, four scenarios have been chosen to analyze the effectiveness of the suggested approach using MATLAB. The number of vehicles being serviced, the overflow rate, the waiting time, and the reduction in overall waiting time are all factors.

## 5. Results and Discussions

**Figure 2:**
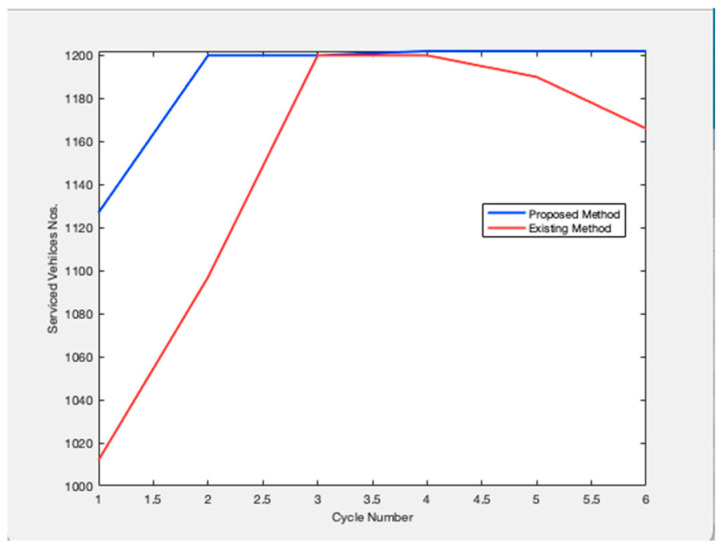
Number of Serviced Vehicles.

**Figure 3:**
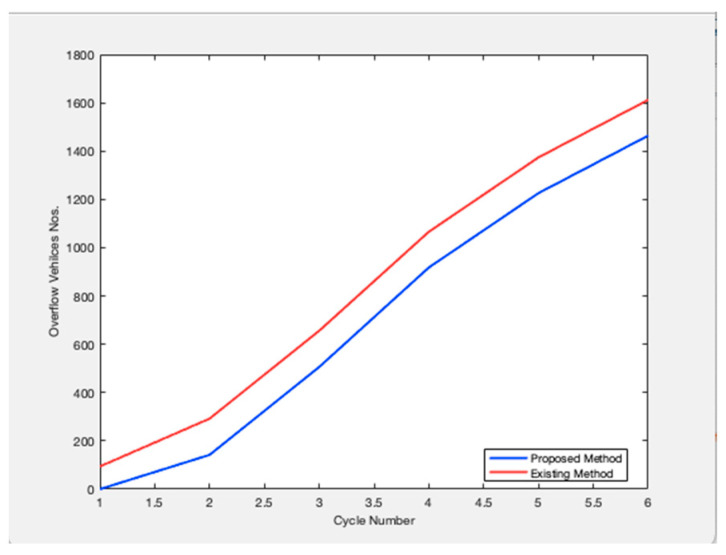
Overflow Vehicle Number.

**Figure 4:**
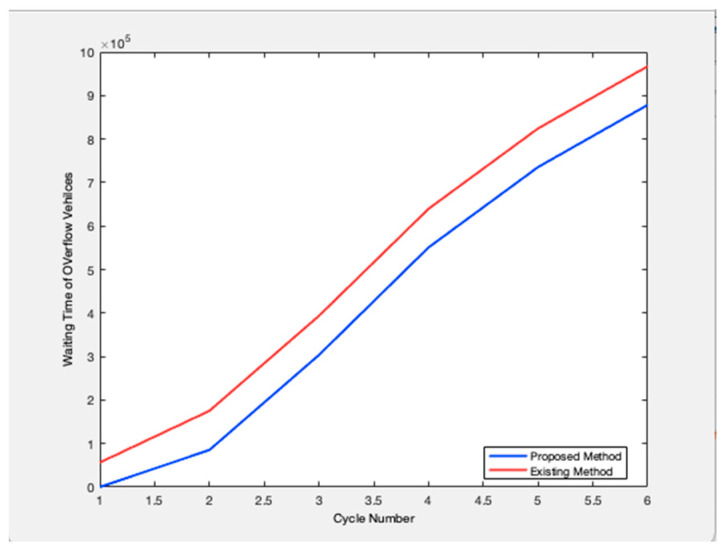
Waiting time overflow.

**Figure 5:**
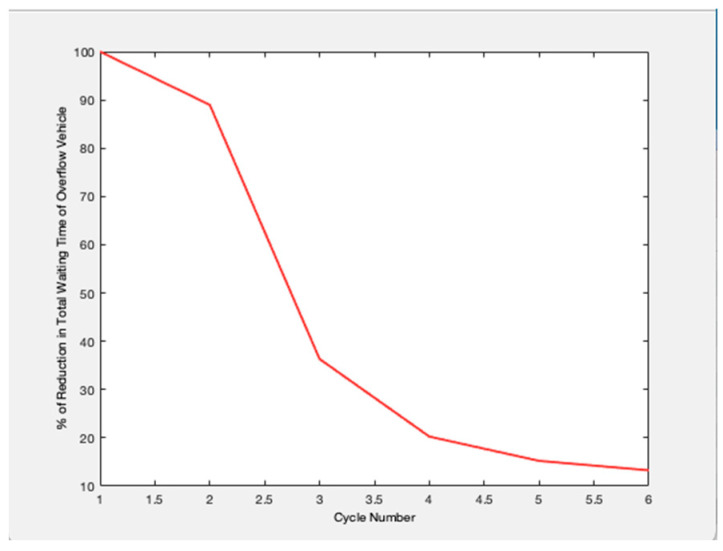
Reduction in Total Waiting time overflows.

Future traffic management plans are generated and evaluated simultaneously over both space and time by the approach. It is intended for dynamic situations where the volume of traffic changes from cycle to cycle. Based on changing traffic conditions, the method provides a unique control for each cycle. The management plans to employ a dynamic process that explicitly takes demand and present and projected queue lengths into account when designing signal control settings. On system links, queue creation, and dissipation are dynamically managed using the signal control parameters. To create management plans to meet specific system performance goals, the traffic management algorithm can be customized. Figure 2 shows the relationship between the cycle length and the number of service vehicles. Any road traffic signal system’s primary goal is to increase throughput, or the volume of cars being served to cross at the intersection. Data on the number of arriving vehicles is gathered for all potential directions, and one cycle length is defined as the total number of arriving vehicles in all directions. In this case, cycle times and the number of vehicles being serviced are connected. The suggested model outperforms the traffic signal already in use. Because this metric measures the effectiveness of the traffic signal system, Figure 3 examines the overflow rate. Figure 4 shows the variation in the length of the traffic vehicle backlog from the proposed and existing models. Any form of traffic signal system absolutely must have this parameter. Observed that the proposed model is superior to the current paradigm in terms of effectiveness. A road traffic light is defined as an effective model if there is less waiting time and an ineffective model if there is more waiting time. As shown in Figure 5, the suggested model outperforms the current approach in terms of time savings. The percentage of improvement in the overall waiting time of overflow vehicles at a significant level is observed.

## 6. Conclusions

In this work, the waiting time for vehicular traffic is examined using a signal distribution control algorithm. The results of the Conventional and the proposed methods are compared and it is observed that the proposed method provided better assistance for road traffic management systems with the range of 20.49%. The method accurately reflects the current state of road traffic. The advantages of dynamic signal schemes over conventional ones and proposed the computation strategy to build a dynamic signal scheme are described. The model assesses the suggested improvement in the technique’s performance. Our analysis shows that a predictive signal strategy based on an autonomously produced program that depends on the current arrival can produce superior results based on some weighted averages.

## Figures and Tables

**Table 1:** Data Collection.

Date	Time	Red Signal Duration/Cycle	Green Signal Duration for the Direction	N-S	W-E	S-N	E-W	Total No. of Vehicles
2 April 2023	7.00–7.10	450	600	112	196	151	113	572
2 April 2023	7.10–7.20	450	600	151	226	172	122	671
2 April 2023	7.20–7.30	450	600	182	244	202	154	782
2 April 2023	7.30–7.40	450	600	196	241	202	166	805
2 April 2023	7.40–7.50	450	600	184	222	191	157	754
2 April 2023	7.50–8.00	450	600	179	208	183	149	719
3 April 2023	7.00–7.10	450	600	279	302	247	221	1049
3 April 2023	7.10–7.20	450	600	282	302	254	221	1059
3 April 2023	7.20–7.30	450	600	277	289	245	219	1030
3 April 2023	7.30–7.40	450	600	281	299	256	211	1047
3 April 2023	7.40–7.50	450	600	278	299	245	213	1035
3 April 2023	7.50–8.00	450	600	249	278	202	177	906
3 April 2023	8.00–8.10	450	600	116	179	129	111	535
4 April 2023	7.00–7.10	450	600	191	162	177	141	480
4 April 2023	7.10–7.20	450	600	189	202	166	133	501
4 April 2023	7.20–7.30	450	600	188	221	166	142	529
4 April 2023	7.30–7.40	450	600	187	211	203	167	581
4 April 2023	7.40–7.50	450	600	195	233	181	176	590
4 April 2023	7.50–8.00	450	600	189	241	193	159	593
5 April 2023	7.00–7.10	450	600	141	155	123	100	519
5 April 2023	7.10–7.20	450	600	129	145	110	91	475
5 April 2023	7.20–7.30	450	600	123	139	119	89	470
5 April 2023	7.30–7.40	450	600	131	165	129	88	513
5 April 2023	7.40–7.50	450	600	133	166	98	88	485
5 April 2023	7.50–8.00	450	600	124	109	89	81	403
5 April 2023	8.00–8.10	450	600	109	187	154	97	547
6 April 2023	7.00–7.10	450	600	146	171	123	95	581
6 April 2023	7.10–7.20	450	600	134	167	112	91	568
6 April 2023	7.20–7.30	450	600	136	166	111	84	592
6 April 2023	7.30–7.40	450	600	129	141	134	111	597
6 April 2023	7.40–7.50	450	600	133	129	113	86	537
6 April 2023	7.50–8.00	450	600	123	131	104	92	526
7 April 2023	7.00–7.10	450	600	144	182	119	99	509
7 April 2023	7.10–7.20	450	600	144	188	122	92	517
7 April 2023	7.20–7.30	450	600	139	171	118	107	518
7 April 2023	7.30–7.40	450	600	135	179	117	103	524
7 April 2023	7.40–7.50	450	600	141	123	110	78	433
7 April 2023	7.50–8.00	450	600	136	119	94	82	424

## Data Availability

Not applicable.
